# Supplementary material for: Diversity and Regulation of S-Adenosylmethionine Dependent Methyltransferases in the Anhydrobiotic Midge
Source: Insects. 2020 Sep 16;11(9):634. doi: 10.3390/insects11090634 (PMC7565475; doi:10.3390/insects11090634)
Supplement: Supplementary file 1 [file insects-11-00634-s001.zip › Sup.Figures.docx]

Supplementary Figures


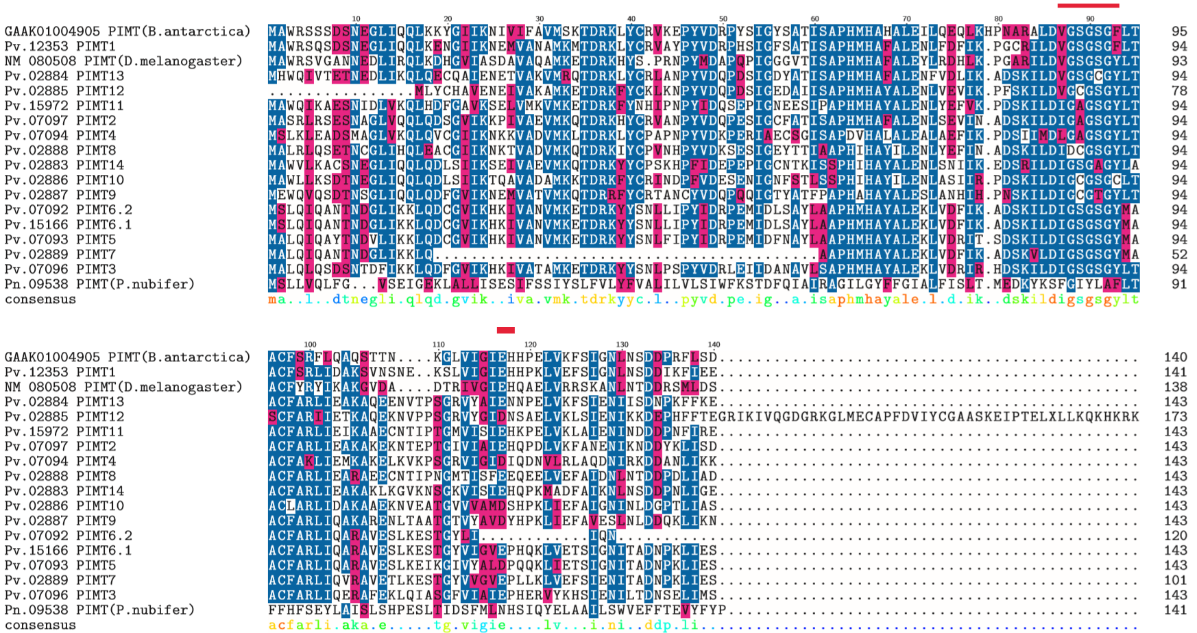


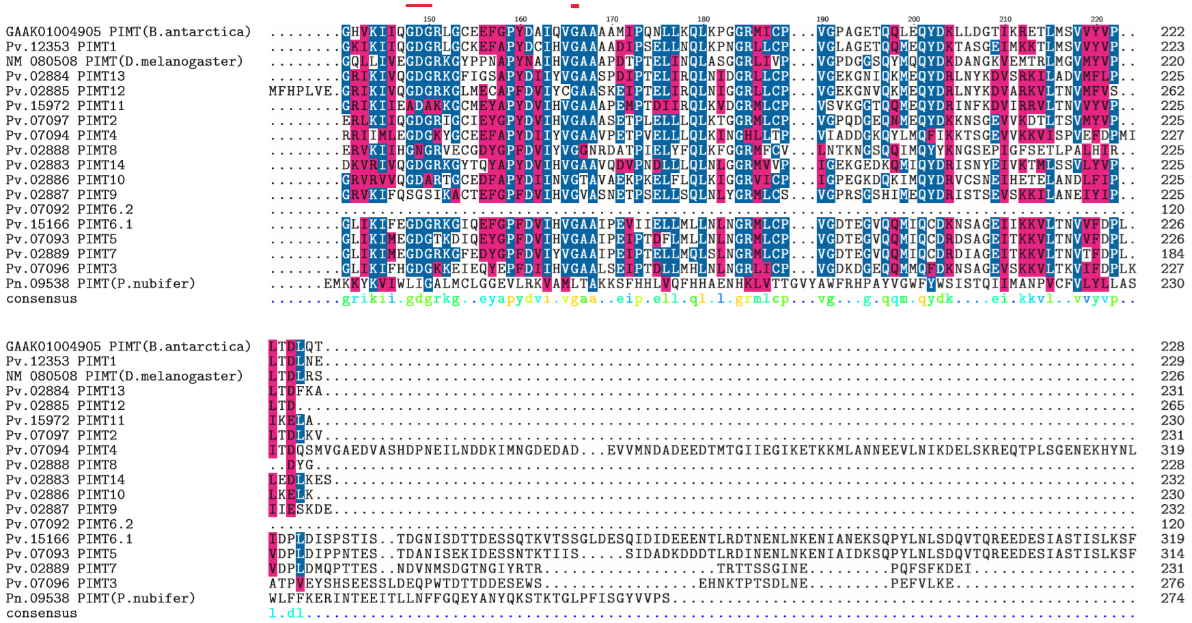


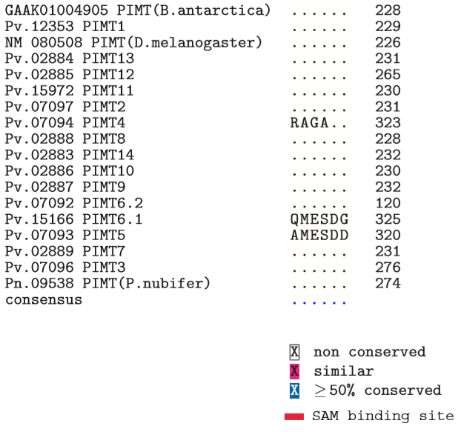


Figure 1. Protein sequences alignment for PIMTs. Red line defines SAM binding site.


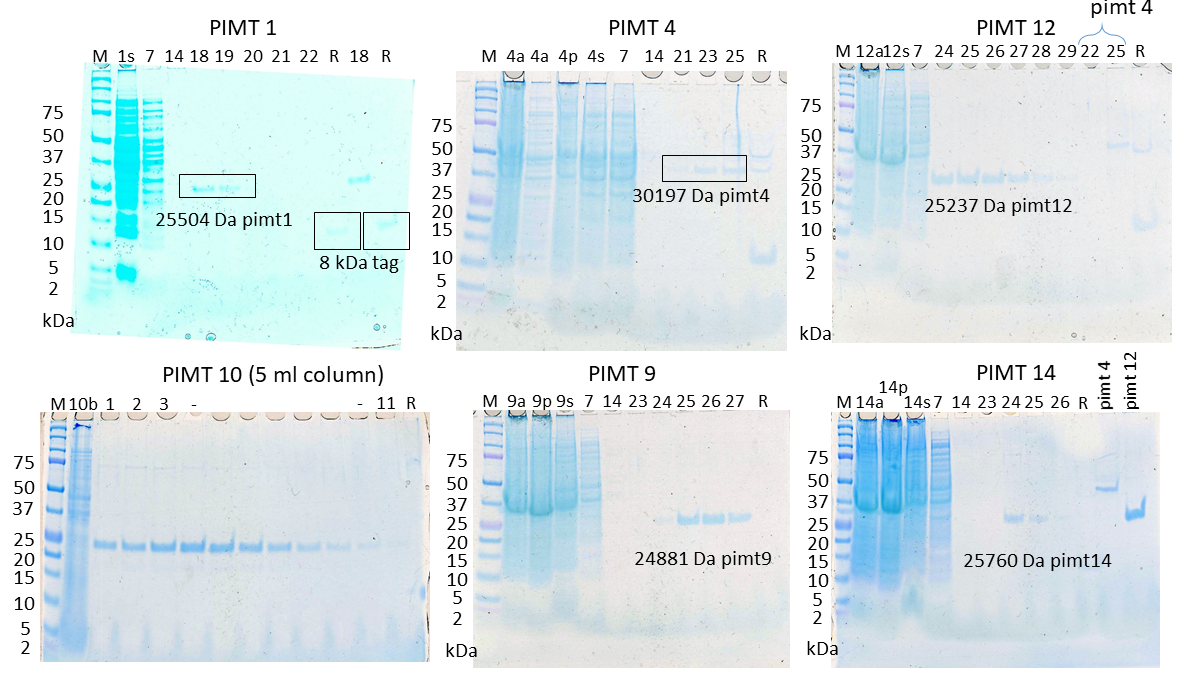


Figure 2. SDS-PAGE for PIMTs (1 and 5 ml column). M – marker, a – after cell destruction (BugBuster), p – pellet fraction after centrifugation, s – supernatant, 1-27 – chromatography fractions, R – fraction after column elution with Regeneration Buffer.


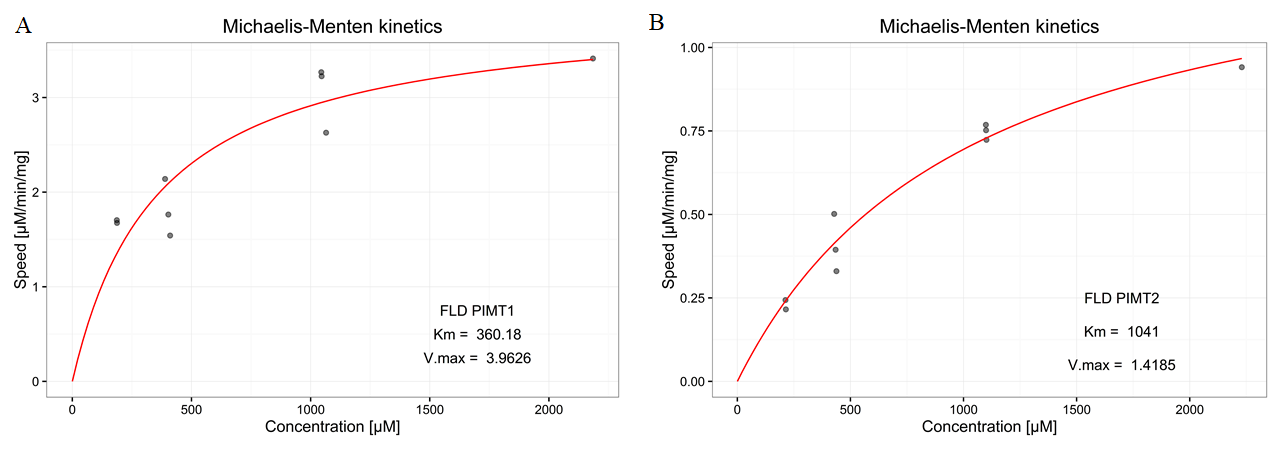


Figure 3. Michaelis-Menten kinetics for PIMT1 and PIMT2 recombinant proteins of *P.vanderplanki*. Estimation based on fluorescent detector data.
